# Data-driven ANN-based visual decoding enables unsupervised functional alignment

**DOI:** 10.1038/s42003-025-09486-7

**Published:** 2026-01-08

**Authors:** Xin-Ya Zhang, Hang Lin, Zeyu Deng, Markus Siegel, Earl K. Miller, Gang Yan

**Affiliations:** 1 Center for Interdisciplinary Studies and Department of Physics, School of Science, Westlake University, Hangzhou, People’s Republic of China; 2 School of Physical Science and Engineering, Tongji University, Shanghai, People’s Republic of China; 3 Shanghai Research Institute for Intelligent Autonomous Systems, State Key Laboratory of Autonomous Intelligent Unmanned Systems, MOE Frontiers Science Center for Intelligent Autonomous Systems, and Shanghai Key Laboratory of Intelligent Autonomous Systems, Tongji University, Shanghai, People’s Republic of China; 4 Department of Computer Science, Lyle School of Engineering, Southern Methodist University, Dallas, TX USA; 5 Department of Neural Dynamics and Magnetoencephalography, Hertie Institute for Clinical Brain Research, University of Tübingen, Tübingen, Germany; 6 Centre for Integrative Neuroscience, University of Tübingen, Tübingen, Germany; 7 German Center for Mental Health (DZPG), Tübingen, Germany; 8 The Picower Institute for Learning and Memory, Massachusetts Institute of Technology, Cambridge, MA USA; 9 Department of Brain and Cognitive Sciences, Massachusetts Institute of Technology, Cambridge, MA USA

**Keywords:** Intelligence, Neural decoding

## Abstract

Artificial neural networks (ANNs) offer a data-driven approach to reveal brain regional functions without explicit supervision. Here, we demonstrate that an ANN trained to decode visual stimuli from multi-unit spiking activity in monkeys, can not only reconstruct complex and dynamic scenes, but also spontaneously align with canonical cortical visual functions. Without any region-specific functional priors, the model identifies key brain areas associated with shape, color, and motion processing. We provide strong evidence that, despite low train-test dataset correlation at the recording-site level, the ANN-based model is able to learn task-relevant representations embedded at a high-dimensional population level and achieve reliable decoding performance. Furthermore, by inverting the architecture and retraining, we show that the same network can predict region-specific spiking patterns from visual input, indicating a reciprocal relationship between encoding and decoding. These findings shed light on ANN-based visual decoding as a powerful framework for unsupervised functional alignment in neural systems.

## Abstract (web summary)

A data-driven artificial neural network model decodes dynamic visual stimuli from monkey multi-unit spiking activity, reconstructing shapes, colors, and motion with high fidelity. It spontaneously aligns with canonical cortical visual functions without region priors, and its inverse architecture predicts neural spiking from visuals, revealing reciprocal encoding-decoding in neural systems.

## Introduction

Artificial neural networks (ANNs) have emerged as powerful tools for understanding complex systems, particularly in neuroscience^1–4^. Their learning mechanisms offer the potential to radically enhance our understanding of neural systems^5–11^. In recent years, advancements in ANNs have driven growing interest in mind reading and neural encoding^12–15^. Within this field, vision-related questions, especially decoding visual information from spiking activity, present a formidable challenge. This task involves scalar, discrete spike frequencies into continuous visual representations that capture complex features such as color schemes, brightness, and shape. Conceptually, it requires mapping a richly structured and continuous output space from sparse and noisy inputs, often under conditions of low train-test dataset correlation. Despite these difficulties, ANN-based decoding holds promise due to its ability to uncover functionally relevant and latent correspondences between visual stimuli and neural recordings.

While diverse architectures of ANNs have been proposed, interpretability remains crucial in determining their adequacy as brain models^16–19^. A fundamental question persists: Does the ANN-based model learn like a brain does? Previous studies have found some convergence in task-oriented attention^20,21^, such as implicit attention in convolutional neural networks (CNNs)^22^ and long short-term memory networks (LSTMs)^23^. However, it remains unclear whether ANNs trained solely to decode visual stimuli, without any region-specific functional priors, can develop representations that spontaneously align with canonical functional properties of the brain (e.g., shape-selective V4, motion-selective MT). This gap extends to the consistency of encoding and decoding models in effectively mirroring region-specific functions, particularly in the domain of brain-computer interfaces.

Here, we analyzed an ideal multi-unit activity (MUA) dataset^24^, which offers superior spatial and temporal resolution compared to functional magnetic resonance imaging (fMRI) data. Unlike previous studies focusing on static, low-dimensional, or single-area representations^25,26^, our approach leverages spiking activity from multiple brain regions in response to dynamic video stimuli, offering a more comprehensive understanding of visual processing. The recordings were obtained from two rhesus monkeys trained on a decision-making task, providing an opportunity to evaluate whether ANNs can extract and decipher spiking patterns, as ANN-based visual reconstruction would be infeasible if the spiking activity were entirely random. During experiments, as the monkeys viewed stimulus videos, trial-by-trial spiking activity was recorded across six brain regions: frontal cortex (lateral prefrontal cortex: PFC and frontal eye fields: FEF), parietal cortex (lateral intraparietal cortex: LIP), and occipitotemporal cortex (inferotemporal cortex: IT, visual area: V4, and middle temporal cortex: MT). Regions LIP, IT, and V4 participate in visual processing^27^, MT is involved in visual motion^28,29^, and cognitive regions PFC and FEF contribute to decision-making processes and influence the integration of visual information^30^. The known functions of these regions serve as ground truths, providing a basis to assess the ability of an ANN-based model to understand intricate neural coding and capture regional functions.

**Fig. 1:**
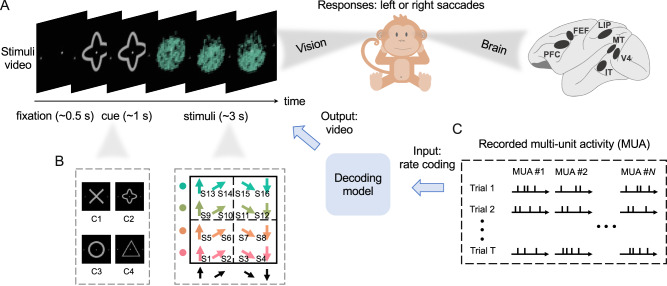
Schematic procedure for decoding stimulus videos from spiking activity recordings. **A** MUA was recorded trial-by-trial in six brain regions when monkeys saw a series of stimulus videos. Stimulus video includes a 0.5-s fixation, a 1-s cue image, and 3-s images of moving stimulus dots. The monkeys were trained on a decision-making task with left and right saccade responses^24^. However, for decoding, the model used rate-coded spiking activity as input and visual stimulus videos as output, without explicitly modeling decision-making, although the spiking data may implicitly carry decision-related information. The cartoon monkey was adapted from Openclipart (Creative Commons Zero, CC0 license), and the monkey brain was adapted from Macauley, S. B. (2020). Zenodo. 10.5281/zenodo.3910249. **B** The combinations of cues and stimuli include a total of 64 types of stimulus videos, from 4 cues (C1 to C4) and 16 stimuli (S1 to S16). Even when the same combination of stimulus video was selected, the initial positions and movement of the stimulus dots varied across trials. **C** MUA neuronal activity was simultaneously recorded at each site (labeled MUA *#N*), capturing the local neuron spiking. Rate coding (spike counts within a 100-ms sliding window) was used to convert MUA data into rate-based spiking activity for the decoding model input.

The stimulus video presented to two rhesus monkeys comprised three main phases: a 0.5-s fixation period, a 1-s cue display, and a 3-s stimulus presentation, as depicted in Fig. 1A. During each trial, the cue image was selected from four possible shapes, while the continuous stimulus consisted of combinations of four possible colors and four possible directions, resulting in a set of 16 types of stimuli (Fig. 1B). Consequently, there were 64 types of stimulus video combinations in total. The initial position and speed of stimulus dots varied from trial to trial. Spiking activity was recorded as discrete spikes (Fig. 1C) when monkeys saw a series of stimulus videos and converted to rate-based activity using a 100-ms sliding window as input to the decoding model.

**Fig. 2:**
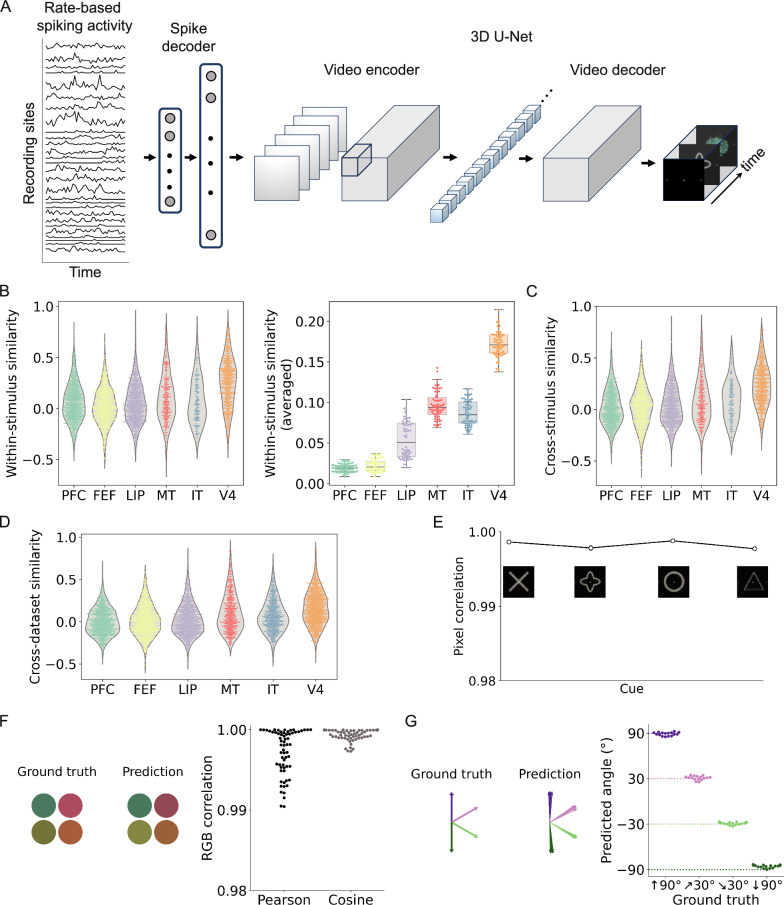
Decoding model architecture, spiking data evaluation, and reconstruction performance. **A** Overview of the decoding model. Spiking data were segmented using a 100-ms sliding window and passed through a two-stage architecture: a two-layer dense spike encoder followed by a 3D U-Net video decoder to reconstruct stimulus frames. **B** Within-stimulus correlations were computed across spiking activities of the same stimulus (e.g., S1 with *n* = 512 independent trial samples). The violin plots show the distribution of similarity values, with the central thick line marking the median and the thin vertical bar indicating the interquartile range (IQR). The averaged similarity for each stimulus condition is summarized with box plots (*n* = 4 × 16 = 64 points), where the central line indicates the median, the box spans the IQR, and whiskers extend to 1.5× IQR (points outside are outliers). Multi-unit rate coding attenuates condition-specific patterns, yet earlier visual areas (e.g., V4 and MT) exhibit slightly higher correlations than frontal regions. Unless otherwise specified, similarity is measured using Pearson correlation. **C** Similarity between spiking activity corresponding to different stimulus conditions (e.g., S1 vs S16) shows a wide range of values within each region (*n* = 512 independent trial samples). Violin plots are as defined in (**B**). **D** Cross-dataset similarity was computed as Pearson correlation of spiking activity between equally sized, randomly sampled subsets from the train and test datasets within each of the six regions (*n* = 64 × 12 independent samples). Violin plots are as defined in (**B**). The overall low correlations imply that any model generalizing across datasets likely captures latent high-dimensional representations rather than superficial correlations. **E** Pearson correlation between pixels of reconstructed and ground truth video frames for different cue shapes, averaged over 16 stimuli per cue (32 × 32 resolution). **F** Pixel-wise correlation (Pearson and Cosine similarity) of RGB values between ground truth and reconstructed stimuli (*n* = 64 independent stimulus samples), showing consistently high reconstruction fidelity. **G** Estimated motion direction extracted from reconstructed videos. Ground-truth and predicted direction vectors are plotted for comparison (*n* = 64 independent stimulus samples).

While previous studies primarily focused on categorizing discrete brain states^31–33^, our approach moves beyond classification to a prediction task with continuous outputs. To achieve this, we drew inspiration from CNNs that have been shown to capture visual representations in a way consistent with biological vision^34–36^. Building on this idea, we present a decoding model that integrates a spike decoder with a 3D U-Net architecture (Fig. 2A), producing visual content (i.e., videos featuring moving colored stimulus dots) directly from spiking activity. The 3D U-Net extends traditional CNNs with a downsampling-upscaling architecture, enabling hierarchical feature extraction across both spatial and temporal dimensions. We then evaluate specific aspects of the predicted videos, including cue shape, stimulus color, and movement. Recent work using generative models, such as diffusion models^37^, while innovative, may be less sensitive to sub-regional data variations due to their inherent noise-based process, potentially limiting their ability to capture the intricacies of neural data. Our approach diverges from establishing direct correspondences between brain regions and ANN layers. Instead, we explore potential functional convergence as a consequence of a data-driven, error-minimization approach.

To foreshadow our results, we provide strong evidence that an ANN-based model trained with an error-minimization objective can spontaneously converge to region-level functional specializations in visual processing without any functional priors. Conventional dimensionality reduction techniques, including both linear methods such as principal component analysis (PCA) and nonlinear methods such as t-distributed stochastic neighbor embedding (t-SNE), show substantial overlap in the trial-by-trial activity across brain regions, suggesting weak region-level separability in low-dimensional projections. In contrast, the ANN model is able to learn and integrate high-dimensional, locally organized yet spatially extended population-level patterns, which induce the emergence of functional specificity. Furthermore, we demonstrate a reciprocal relationship between brain encoding and decoding, as evidenced by the ability of our inverse decoding model to predict spiking activity from visual input with high accuracy.

## Results

### Decoding visual stimulus videos from spiking data with low train-test correlation

We employed a 100-ms-wide sliding window to transform discrete spiking events (Fig. 1C) into a time series of spiking rates (i.e., rate-based spiking activity in Fig. 2A), which served as the input for our decoding model. This process converts the original discrete spikes into a sequence where each element represents the frequency of spiking within each corresponding window. Based on the area and the minimum number of electrode placements across the six brain regions, we utilized 140 recording sites for each trial (PFC: 30 sites, FEF: 30 sites, LIP: 30 sites, IT: 10 sites, V4: 20 sites, MT: 20 sites). To emulate brain function and investigate the relationship between neural spiking activity and visual stimuli, we tailored a decoding model capable of generating corresponding stimulus videos as output (Fig. 2A). This model was trained using the training dataset, and its performance was evaluated on an independent test dataset (see “Methods” section and the results of 5-fold cross-validation in Supplementary Tables 1 and 2). All subsequent analyses were conducted on the test dataset.

To assess the spiking activity, we quantified within-stimulus and cross-stimulus similarities. The within-stimulus similarity measures the correlation of spiking responses under identical stimulus conditions (Fig. 2B), whereas the cross-stimulus similarity quantifies correlations across different stimulus conditions (Fig. 2C). We observed that within-stimulus correlations were generally weak, largely because rate coding loses spike timing information, partially smoothing out stimulus-specific variations. However, if averaging similarity within each stimulus condition, regions V4 and MT exhibited more consistent patterns than other areas (Fig. 2B). We further evaluated cross-dataset similarity to assess how well activity patterns generalize between independently sampled train and test datasets (Fig. 2D). The overall low train-test dataset correlations (i.e., cross-dataset similarity) suggest that models capable of generalizing across datasets are likely capturing latent representations rather than superficial correlations.

The video sequence was segmented to evaluate the cue and stimulus stages separately. For the cue stage (from 0.5 to 1.5 s), we calculated the Pearson correlation *C* of the pixels between reconstructed cues (Fig. 2E) and ground truth (Fig. 1B), regardless of stimulus type (for cross shape: *C* = 0.9986 ± 0.0001, quatrefoil shape: *C* = 0.9978 ± 0.0003, circle shape: *C* = 0.9988 ± 0.0001, and triangle shape: *C* = 0.9977 ± 0.0002). For the stimuli stage (from 1.5 to 4.5 s), we extracted four symmetric reference points located at the 2/4 and 3/4 positions along the horizontal and vertical axes of the aggregated stimulus images to minimize the influence of peripheral noise (see Methods for RGB color of stimulus images). These points were used to evaluate the correlation of RGB color values of the stimulus dots by comparing reconstructed videos with the ground truth (Pearson correlation: *C* = 0.9959 ± 0.0023, cosine similarity: *C* = 0.9987 ± 0.0013, Fig. 2F). The motion experiment was configured with angles *θ* = 90°, 30°, −30°, −90°. The predicted motion of stimulus dots across frames was computed using the optical flow method (see “Methods” for movement across stimulus images), resulting in estimates of *θ* = 89° ± 2°, 31° ± 3°, −29° ± 2°, −86° ± 2° in Fig. 2G, respectively. The decoding model achieves high performance across different frequency sampling rates (see Supplementary Movies 1–3). Baseline comparisons are shown in Supplementary Fig. 1, we evaluated multilayer perceptron (MLP) and shallow CNN models (see “Methods” for model details), and found that our decoding model achieved higher similarity and lower reconstruction loss (Supplementary Tables 3 and 4).

### Neural and model representations across video stages and brain regions

**Fig. 3:**
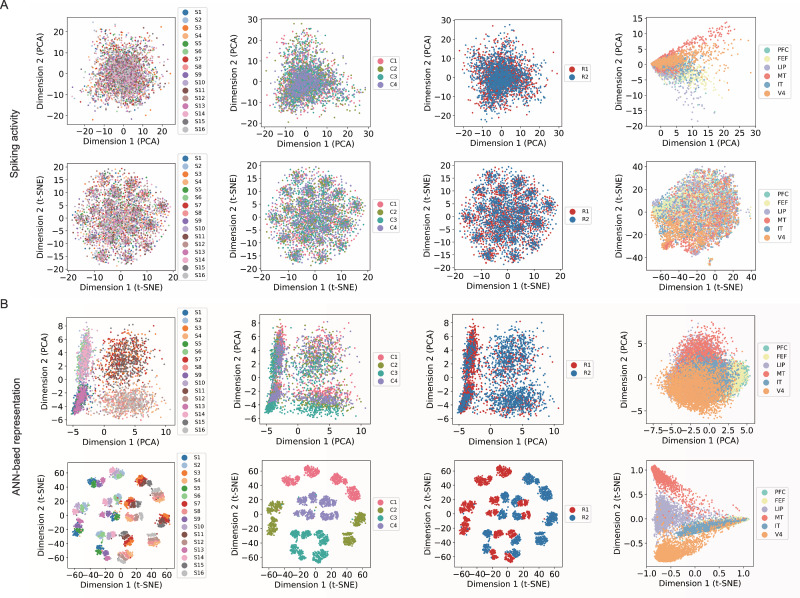
Comparison of spiking activity and ANN-based representations across video stages and brain regions. **A** Dimensionality reduction (PCA, top; t-SNE, bottom) applied to recorded multi-unit spiking activity (*n* = 512 independent trial samples) from six cortical regions (PFC, FEF, LIP, IT, MT, V4). Each column corresponds to the video stage-related category: stimulus (S), cue (C), left/right behavioral response (R), and brain area. The distributions appear largely overlapping across conditions, indicating that the neural data exhibits weak clustering structure in low-dimensional space, regardless of whether the projection is linear (PCA) or nonlinear (t-SNE). **B** The same visualizations for the trained artificial neural network (ANN)-based latent representations. In contrast to spiking data, the model representations exhibit limited separation under linear decomposition (PCA) but become clearly clustered under nonlinear embedding (t-SNE). Distinct clusters emerge by stimulus, cue, and response, particularly evident in MT and V4 for PCA, and in MT, LIP, and V4 for t-SNE, reflecting the emergence of disentangled and structured representations learned by the ANN.

To examine how task- and region-related information is embedded in spiking activity, we applied both linear (PCA) and nonlinear (t-SNE) dimensionality reduction to the recorded MUA data and to the model representations derived from the decoding model. For spiking data (Fig. 3A), both PCA and t-SNE revealed largely overlapping distributions across video stages (cue, stimulus, and response) and brain regions (PFC, FEF, LIP, IT, MT, V4), indicating that neural data exhibit weak clustering structure in low-dimensional space.

In contrast, the ANN-based latent representations, extracted from the intermediate layers of the 3D U-Net (i.e., the blue blocks in Fig. 2A), exhibited a more organized structure (Fig. 3B). The linear PCA projection revealed only partial separation among brain regions, with earlier visual areas (MT and V4) becoming somewhat distinct. The nonlinear t-SNE embedding showed clear clustering by video stage, with distinct clusters emerging for stimulus, cue, and response phases. Regions such as MT, V4, and LIP were well separated, whereas higher-order areas (e.g., PFC and FEF) remained partially overlapping. These results indicate that the decoding model learns to disentangle latent task- and region-related features, capturing nonlinear structure that is not directly evident in the spiking activity.

### Region-level function convergence between the ANN-based model and the brain

**Fig. 4:**
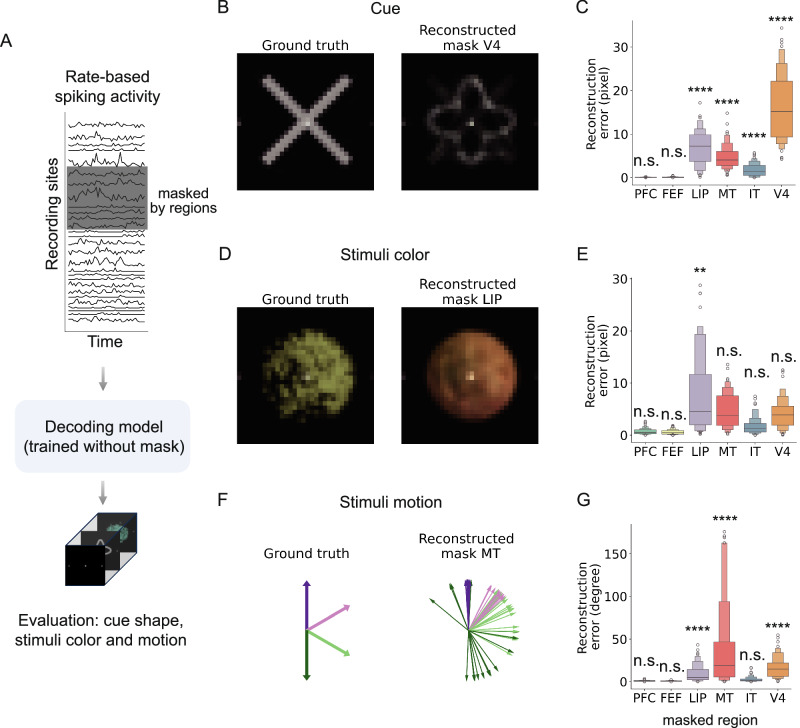
Region-level functional convergence revealed by masking spiking activity. **A** Schematic of the decoding setup with region-specific masking. Spiking activity from six brain regions (PFC, FEF, LIP, MT, IT, V4) was individually masked at test time while the model was trained on the full brain activity. **B**, **D**, **F** Example reconstructions show how masking specific regions degrades decoding accuracy in modality-specific ways: masking V4 impairs shape (**B**), LIP affects color (**D**), and MT disrupts motion direction (**D**). **C**, **E**, **G** Quantitative analysis of reconstruction error across regions. Each boxplot summarizes the reconstruction error over 64 stimulus types for shape (**C**), color (**E**), and motion (**G**), with masking of V4, LIP, and MT leading to the largest errors in their respective modalities. All box plots represent the average difference over 64 types of videos, with the central line indicating the median, the box spanning the IQR, and whiskers extending to 1.5× IQR (points outside this range are shown as outliers). A larger value indicates that masking the specific area has a greater impact on the reconstruction quality. Statistical significance is evaluated using a two-sided *t*-test, comparing each masking condition to the unmasked reconstruction for each individual region (*n* = 64 independent stimulus samples), with statistical significance noted (^*^p < 0.05, ^**^p < 0.01, ^***^p < 0.001, ^****^p < 0.0001).

Can ANNs spontaneously identify region-level functions during visual decoding without any prior functional information? To address this question, we evaluated each brain region by masking its spiking activity in the test dataset, after the decoding model was trained using the complete training data. Here, masking refers to setting the input spikes from a given region to zero in the model, rather than experimentally silencing the brain region, which was not possible with the recorded data. In this approach, we selectively masked spiking activity based on its spatial location (e.g., region) and input the unmasked spiking activity into the trained model to observe the predicted videos (Fig. 4A). To assess the quality of the reconstructed videos, we calculated pixel differences (e.g., RGB values or movement angles across frames) between scenarios with and without spike masks (Figs. 4B–G). A larger reconstruction error indicates a more pronounced impact of the masked region on the quality of visual decoding.

We observed differential contributions of vision-related brain areas to the reconstruction of stimulus videos. Specifically, V4 exhibited a notable impact on the reconstructed shapes of cues, followed by LIP, MT, and IT (mean ± s.d.: V4: 16.42 ± 7.83, LIP: 7.11 ± 4.01, MT: 4.76 ± 2.87, IT: 1.81 ± 1.52). A two-sided *t*-test revealed significant differences in the distributions of pixel (V4: *t*(126) = −16.34, *p* < 0.0001, LIP: *t*(126) = −12.85, *p* < 0.0001, MT: *t*(126) = −11.99, *p* < 0.0001, IT: *t*(126) = −7.42, *p* < 0.0001). PFC and FEF did not significantly affect the reconstruction of cues (see Supplementary Table 5). We identified LIP, MT, V4, and IT as influential in shaping the reconstructed color quality of stimulus dots (mean ± s.d. (RGB value) for LIP: 8.57 ± 8.52, MT: 2.98 ± 2.33, V4: 3.55 ± 2.65, and IT: 1.07 ± 0.93). LIP was the significant influencer towards reconstructed color (*t*(126) = −3.07, *p* = 0.0026, see Supplementary Table 6). Remarkably, MT emerged as a highly influential region for motion direction, with contributions also observed from V4 and LIP (mean ± s.d. (angle degree) for MT: 30.75 ± 40.48, *p* = 0.0014, V4: 15.88 ± 12.61, *p* < 0.0001, and LIP: 9.18 ± 9.06, *p* < 0.0001, two-sided *t*-test statistic in Supplementary Table 7). Multiple-comparison correction was applied, as reported in Supplementary Tables 8–10. Statistical tests confirm that V4 (Fig. 4C), LIP (Fig. 4E), and MT (Fig. 4G) show significant differences compared with other brain regions. These findings highlight the intricate interplay of V4, MT, and LIP in shaping visual perceptions, ranging from visual categorization to motion processing and recognition of complex visual stimuli.

Although the model itself did not implement any decision-making process, the spiking activity used for decoding inherently contained task-related decision signals. Thus, while V4 and MT contributed to shape and motion decoding as expected^38,39^, the strong influence of LIP on color decoding likely reflects decision-related modulation embedded in neural data, consistent with feature-based attention and decision-variable coding in parietal cortex^40,41^. Masking V4 did not produce noisy outputs but instead led the model to reconstruct a combined yet familiar cue. This observation reflects the model’s constrained output space, learned from a limited set of cues and stimuli, explaining why the model output remains blended rather than degenerate.

### Decoding dynamic images from individual brain areas

The results of visual decoding from spiking activity across multiple brain regions demonstrate impressive performance. However, a crucial question remains: Is there redundancy in the brain for visual stimuli in individual areas? In simpler terms, can the stimulus video be decoded from individual areas? Here, we trained the decoding model using spiking activity from limited areas and then evaluated the decoding performance of reconstructed visual videos from individual brain regions. Specifically, we used the training dataset extracted from limited areas for model training, and the corresponding test dataset from the same limited areas for evaluation.

**Fig. 5:**
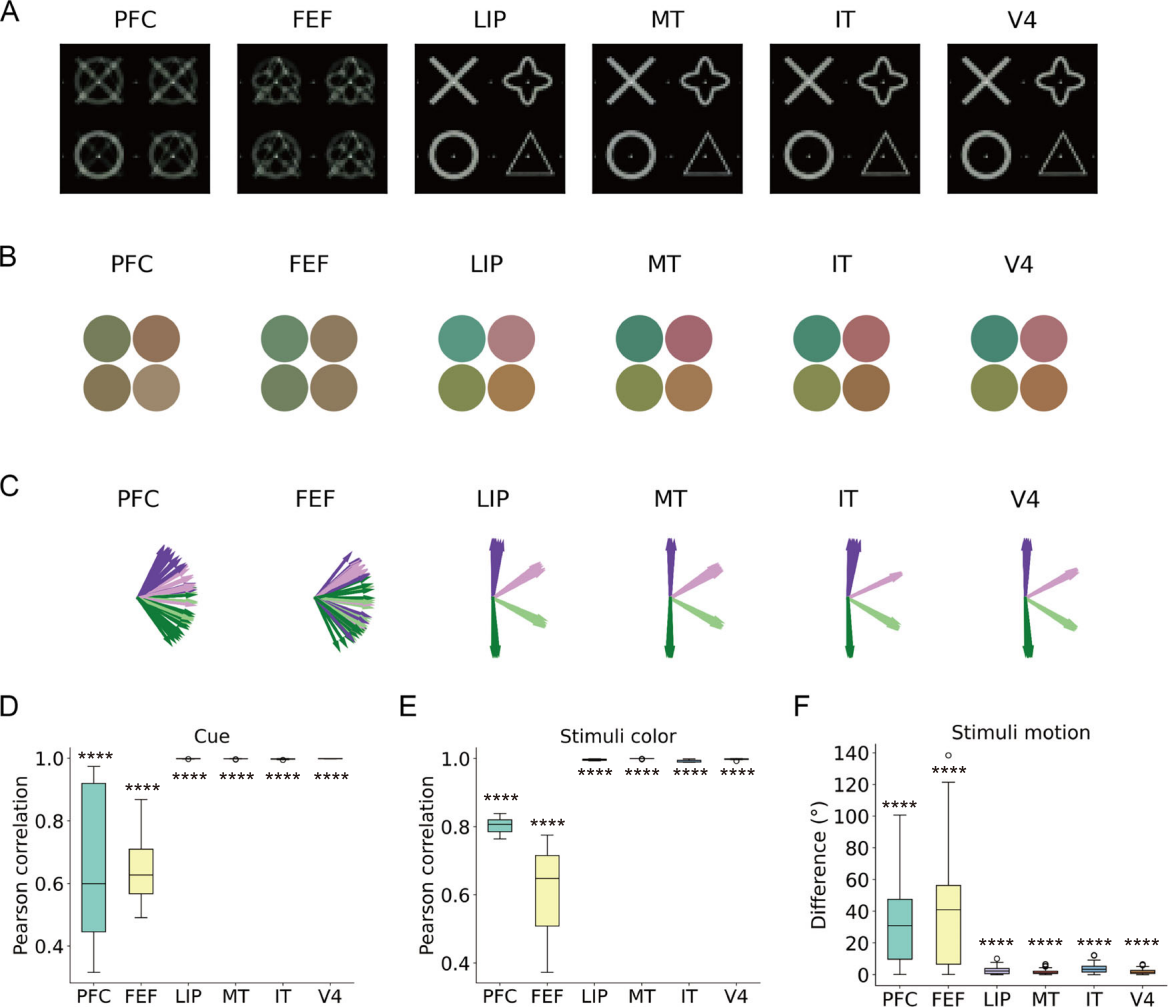
Decoding visual features from activity in individual brain regions. **A** Reconstructed cue based on spiking activity in six brain regions individually. A high-quality reconstruction would showcase distinct cues, such as those from individual regions LIP, MT, IT, or V4. **B** The color of stimulus dots extracted from the reconstructed stimuli. The left side features a greenish color scheme, while the right side is reddish. The ground truth comprises four distinct colors, as observed in the reconstruction from LIP, MT, IT, or V4. **C** The motion of stimulus dots is estimated from the stimulus video across frames. A high-quality reconstruction would reveal four distinct directions, as shown from LIP, MT, IT, and V4. **D** Pearson correlation between the ground truth and the reconstructed cues (**A**). Each box represents the average correlation over 64 types of videos. A higher correlation value indicates a stronger resemblance to the ground truth. **E** Similar evaluation for RGB value based on reconstructed stimuli (**B**). Higher correlation indicates better performance. **F** The angle difference between the ground truth and the reconstructed stimulus motion (**C**). A smaller difference indicates superior performance. To evaluate whether the decoding performance exceeded chance levels, a two-sided *t*-test was used to compare the real performance (*n* = 64 independent stimulus samples) against a null distribution generated from 100 predictions based on Gaussian random inputs. Asterisks denote significance levels (^*^p < 0.05, ^**^p < 0.01, ^***^p < 0.001, ^****^p < 0.0001). For all box plots (**D**–**F**), the upper and lower boundaries of the box denote the first and third quartiles, the horizontal line inside the box denotes the median, and points beyond 1.5× the IQR are shown as outliers.

As depicted in Fig. 5, the stimulus videos cannot be well-reconstructed if only the spiking activity in the PFC or FEF is utilized, as indicated by lower correlation and higher motion difference. In contrast, each of the other four regions can be leveraged to reconstruct videos that exhibit a high correlation with the ground truth (mean: LIP: *C* = 0.9986, MT: *C* = 0.9985, IT: *C* = 0.9977, V4: *C* = 0.9986, Supplementary Table 11). While PFC and FEF appear to capture the coarse color scheme due to the model’s constrained output space, they lack the ability to generate refined color details (Fig. 5B). Spiking activity in individual LIP, MT, IT, or V4 can reconstruct detailed colors (mean: LIP: *C* = 0.9967, MT: *C* = 0.9997, IT: *C* = 0.9930, V4: *C* = 0.9982, Supplementary Table 12). Regarding motion, as shown in Fig. 5C, PFC and FEF are less effective in predicting four directions (i.e., with a high error bar, PFC: s.d. = 25.32°, FEF: s.d. = 34.88°, Supplementary Table 13). We also performed 100 independent reconstructions from random Gaussian noise and compared them with reconstructions generated using activity from each brain region (Supplementary Tables 11–13). The results indicate that, although PFC and FEF cannot fully reconstruct the stimuli, they still contain partial information about the cues or stimuli beyond chance levels.

### Encoding and decoding are reciprocal processes

**Fig. 6:**
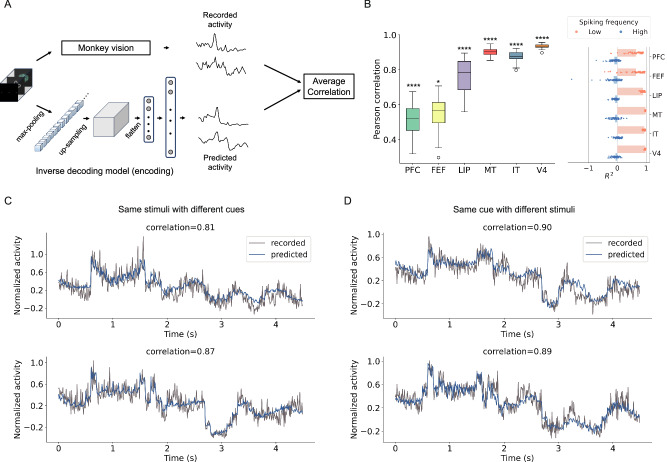
Performance of the inverse decoding model. **A** Recorded activity refers to the spiking recordings when monkeys watched videos, and predicted activity is the output of the inverse decoding model. The similarity between the recorded and the predicted activities is quantified by Pearson correlation. **B** Pearson correlation for each of the six brain regions. A higher correlation indicates a closer resemblance between predicted and recorded activities in the specific region. The central line in the box plots denotes the median, the upper and lower boundaries of the box denote the first and third quartiles, and points beyond 1.5× the IQR are shown as outliers (circles). Statistical significance was assessed against a null distribution generated by predicting activity from Gaussian noise in individual regions (*n* = 64 independent stimulus samples). The null distribution was obtained from 100 noise-based predictions, and significance was assessed using a two-sided *t*-test, with asterisks indicating significance levels (^*^p < 0.05, ^**^p < 0.01, ^***^p < 0.001, ^****^p < 0.0001). The *R*^2^ scores are shown for low- and high-frequency components of both recorded and predicted spiking activity, obtained by decomposing the activity using a Butterworth filter. Each bar represents the mean value of *R*^2^ for the corresponding region (*n* = 64 stimuli) and frequency band. **C** Examples of recorded (gray) and predicted (blue) activities in region IT. The monkey was shown the same type of stimulus combination but with different cues. **D** Examples of recorded (gray) and predicted (blue) activities in region IT. The monkey was shown different stimulus combinations, but the same type of cue.

Having demonstrated the ability of an ANN-based model to decode dynamic images from brain spiking activity, we next investigated whether encoding and decoding are reciprocal processes. While previous studies have employed distinct models for decoding and encoding brain activity^42,43^, we adopted a more direct approach by exploring whether an inverse version of the decoding model could effectively encode visual stimuli. Specifically, we inverted the model architecture (i.e., a 3D U-Net followed by a spike decoder, as detailed in Methods) and re-trained it. This approach allowed us to generate spiking activity for each cue or stimulus by reversing the input-output relationship, mirroring the inverse of the decoding process (Fig. 6A). We then compared the predicted spiking activity to the recorded activity averaged within each brain region.

Figure 6B shows that the correlation between the predicted and the true spiking activity is high for vision-related regions LIP, MT, IT, and V4 (mean: LIP: *C* = 0.7671, MT: *C* = 0.9016, IT: *C* = 0.8772, and V4: *C* = 0.9352, see Supplementary Table 14). Such high performance was achieved by the inverse decoding model, suggesting that decoding and encoding are indeed reciprocal. In contrast, the correlation is relatively lower for PFC and FEF (mean: PFC: *C* = 0.5146 and FEF: *C* = 0.5476), which are less relevant to visual information processing. Moreover, we conducted 100 independent tests by using random videos containing randomly generated pixel values as input, finding that, even in vision-unrelated areas such as PFC and FEF, the correlation is significantly higher than random prediction (PFC: *t*(162) = 7.624, *p* < 0.0001 and FEF: *t*(162) = 2.078, *p* = 0.0391, see Supplementary Table 14). This result suggests that PFC and FEF may encode partial information of vision, but such encoding appears to be limited, possibly due to functional constraints. Figure 6C, D displayed examples of the spiking activity, both recorded and predicted, under different stimuli and cues.

To further examine how the predicted activity captures the temporal properties of spiking signals, we decomposed both ground-truth and predicted activity into low- and high-frequency components using a Butterworth filter^44^. We then computed the coefficient of determination (*R*^2^) for each frequency band in individual regions (Fig. 6B). The prefrontal region (e.g., PFC) exhibited weaker correspondence with the ground-truth activity, showing relatively low *R*^2^ values for both frequency bands. In contrast, sensory regions such as V4 and MT demonstrated higher *R*^2^, particularly in the low-frequency band. This observation suggests that the reduced predictability in PFC reflects its more high-frequency and variable spiking patterns (Supplementary Fig. 2), consistent with its involvement in higher-order or context-dependent processes beyond direct visual stimulus representation.

## Discussion

Our study demonstrates that the ANN-based model can reliably decode dynamic visual stimuli from spiking recordings in non-human primates (Fig. 1). Despite the absence of high train-test dataset correlation, and substantial regional overlap observed through both linear and nonlinear dimensionality reduction analyses, the ANN-based model successfully reconstructs fine-grained visual features, including shape, color, and motion direction (Fig. 2). The ANN is capable of learning these latent representations and mapping them to visual content (Fig. 3), indicating that it may capture the core computational principle underlying visual processing in the brain.

In addition to training our model using data from multiple brain areas, we investigate the reconstruction performance using data from individual regions. When training the model using data from an individual region, the model relies solely on that region, yet still achieves comparable performance in most areas. This indicates that a large portion of stimulus-related information lies in a shared subspace redundantly expressed across cortical regions, except for PFC and FEF. However, when trained on all regions, removing a key region reduces performance, reflecting regional specialization alongside redundant signals. This occurs because, in addition to the shared subspace, the model trained on multi-region data learns to exploit region-specific complementary features during joint training. As a result, multi-region integration enables the decoder to use distinct and non-overlapping information from each area, and masking a region disrupts these complementary contributions. These results suggest that spiking activity exhibits population patterns that are locally organized yet spatially extended, allowing the ANN to integrate information across regions for visual processing.

Moreover, we invert the architecture of the decoding model to predict spiking activity given stimulus videos. This approach shows that decoding and encoding are indeed reciprocal processes. The impact of different brain regions observed in the decoding process remained consistent in the encoding process. While visual stimuli could be encoded across multiple brain regions, performance was notably lower when encoding dynamic images from vision-unrelated regions, particularly higher-order areas. This reduction is likely due to their limited representation of both low- and high-frequency components of the spiking activity (Fig. 6). These findings indicate the bidirectional nature of the relationship between brain decoding and encoding, and highlight the potential limitations of brain encoding in higher-order areas.

Compared to baseline models, which either lose spatial or temporal information (see Methods, Supplementary Fig. 1 and Tables 3 and 4), the 3D U-Net is able to leverage both temporal and spatial information, thereby achieving better performance. Other potential decoding models, such as generative adversarial networks (GANs) and stable diffusion models, are effective in generating realistic images from noisy or incomplete data, this very effectiveness can pose challenges in providing clear explanations or interpretations of which features contribute to their generation^45^. To advance scientific discovery using ANNs, it is crucial not only to prioritize the ability of reconstruction but also to unravel the interpretability of ANNs. This dual focus ensures that ANNs not only generate outputs but also provide insights into how and why those outputs are produced.

Our work focused on the correspondence between visual stimuli and spiking activity without explicitly modeling decision-making or eye movements, although these processes likely influenced the recorded data. Region LIP integrates sensory evidence and encodes choice- and attention-related signals^40,41^, indicating that spiking data could capture both stimulus-driven and higher-order cognitive modulations. Thus, the decoding model trained on such activity likely learns a mixture of sensory and decision-related representations. LIP’s contribution to color decoding may therefore arise from its role in integrating sensory features with attentional or decision-related components, not because LIP encodes color per se, but because higher-order regions can carry task-modulated activity that indirectly correlates with visual features.

Unlike the underutilization of PFC and FEF observed here, prior work^23^ reported that ANN-based models exhibit strong representational alignment with these regions under high cognitive demands, particularly when saccadic decision-making is explicitly required during training. These results suggest that under varying task demands, ANN-based models can develop distinct region-specific functions, with high correspondence to vision-related areas when visual processing is emphasized. Future work could extend this approach to assess whether similar task-driven convergence emerges across species or behavioral contexts, offering a promising avenue to validate functional interpretations derived from ANN-based models.

In both visual decoding and encoding tasks, the ANN-based model was trained without any explicit information about the functional roles of cortical regions. Nevertheless, distinct region-specific contributions emerged spontaneously. This unsupervised emergence of functional specialization resembles self-organization processes in the brain^10,46,47^, where regional functions arise from interactions among neurons rather than explicit supervision. This ability highlights the potential of ANN-based models not only as engineering tools but also as instruments for scientific discovery. By leveraging high-dimensional population activity, ANNs capture latent computational principles embedded in neural data that are often inaccessible to conventional analytical methods. These findings support the use of ANN-based approaches in neuroscience and suggest that such models could uncover previously unrecognized relationships between neural dynamics and cognitive processes, offering avenues for understanding brain function^48^.

## Methods

### MUA

MUA data were obtained from a prior study, ref. ^24^, in which MUA was recorded from a total of 2694 sites (1753 and 941 from the two rhesus monkeys) across 48 recording sessions. All procedures performed on the two rhesus monkeys (one male, one female) followed the guidelines of the USDA and the Massachusetts Institute of Technology Committee on Animal Care. All procedures were approved by the Massachusetts Institute of Technology Committee on Animal Care. Each monkey was implanted with a titanium head bolt to immobilize the head. Recordings covered six cortical regions: the dorsolateral prefrontal cortex (dlPFC, 1020 sites), FEF (532 sites), lateral intraparietal area (LIP, 807 sites), middle temporal area (MT, 123 sites), inferior temporal cortex (IT, 57 sites), and visual area V4 (155 sites). Note that each session involves some electrode replacements, leading to a large number of recording sites. The stimuli in each trial were randomized. Recordings were performed using Epoxy-coated tungsten electrodes. Electrodes were advanced simultaneously in pairs or triplets, with penetrations typically angled relative to the cortical surface. This procedure did not aim for a specific layer or fine-tune the positioning for specific cells. Thus, recordings were from all cortical layers with no bias for a specific depth.

To ensure balanced regional sampling for decoding, we randomly selected a pooled subset of 140 representative sites (PFC: 30, FEF: 30, LIP: 30, IT: 10, V4: 20, MT: 20). The electrode sites were not fixed across sessions due to electrode replacements. The lower bound was determined by the region with the fewest available sites (IT), allowing all regions to contribute comparably without discarding a substantial portion of data from regions with denser coverage. Importantly, decoding performance did not scale systematically with the number of sites, indicating that the observed effects primarily reflect functional content rather than the electrode count. To ensure fairness across conditions, we extracted *T* = 512 trial samples for each stimulus type. Specifically, spiking activity was constructed from trials within the same stimulus condition by randomly choosing sites within each region. For model input, spiking activity was represented using rate coding (i.e., spike counts) computed with a 100-ms sliding window. The resulting features were then standardized (transformed to have zero mean and unit variance). Note that standardization differs from normalization, which scales data to a fixed range (e.g., between −1 and 1).

### Task details

Two rhesus monkeys, a female named Paula, and a male named Rex, underwent training for a categorization task. The animals had to respond with one direct saccade that categorized either the color (red vs green) or motion direction (up vs down) of the stimulus based on the task cue. For correct responses, the animals were rewarded with apple juice. After years of training, spiking activity was recorded in six brain regions. In each recording session, all pairs of regions were recorded simultaneously at least once. Each session comprised multiple trials, where each trial consisted of a sequence of animated images, including a fixation (lasting 0.5 s), a cue (lasting 1 s), and stimuli (lasting 3 s). The cue could be one of four types: cross, quatrefoil, circle, or triangle shapes. After the cue period, the cue was switched off, and stimuli appeared at the center of the fixation spot. Spiking activity was recorded across brain areas simultaneously throughout the videos. The videos featured a static fixation point and cue images, while the stimulus images were dynamic, consisting of 400 moving dots.

### Stimuli video

Each stimulus video consisted of three segments: a fixation period (0–0.5 s), a cue period (0.5–1.5 s), and a stimulus presentation period (1.5–4.5 s). Depending on the task cued at the beginning of each trial, the monkeys categorized either the color (red vs green) or motion direction (up vs down) of the stimulus and reported their perception with a left or right saccade. Our analysis focused on the visual stimuli and corresponding neural spiking, independent of the saccades. Each recording trial used a stimulus consisting of randomly positioned colored dots moving coherently. The stimuli set included four possible colors and four possible motion directions (Fig. 1). This design allowed for 16 color-motion combinations, with the random initial dot positions ensuring variability in each stimulus. For detailed specifications of the stimulus images, please refer to the Supplementary Information.

Each trial was initiated when the monkey fixated on a point at the center of the screen and required fixation within 1. 2° of visual angle from the fixation point, which was maintained throughout. Following a fixation period (500 ms), the monkey was presented with a visual task cue lasting 1000 ms. The cues, depicted in Fig. 1B, consisted of one of four distinct gray shapes, each approximately 1. 5° in visual angle diameter, and were centrally presented on the fixation spot. After the cue period, the cue disappeared, and stimulus dots appeared at the center of the fixation spot. These were images of colored dots with random initial positions, all moving synchronously in a specific direction. The random dots appeared within a circle centered at the fixation point. All stimuli were 100% coherent, combining one of four possible colors and one of four possible motions. Due to the randomness of position and velocity, the stimuli exhibited fluctuation in color direction within a circular region when averaged or integrated. Hence, we hypothesized that the ANN-based model is capable of learning averaged color and motion across trials.

### Decoding model

To reconstruct chromatic and continuous vision from spiking data, we employed a decoding model, consisting of a spike decoder denoted as **f**, and a 3D U-Net (i.e., video encoder-decoder), as **g** and **h**, respectively. These components work cohesively to decode spikes into hidden features and then generate the corresponding video output.

The spike decoder utilizes a two-layer fully connected neural network to transform spiking data $x\in {{\mathbb{R}}}^{N\times N}$ to images using the Unflatten operation,

1 $$\widehat{x}={{{\bf{f}}}}(x).$$

where *N* = 140 sites and *N* = 45 time points corresponding to a 100-ms bin within 4500 ms. Batch normalization and ReLU activation between layers were implemented to ensure effective training and introduce non-linearity, aiding the transformation from spiking data to images.

CNNs are inspired by human visual neurons^49^. The 3D U-Net is a video encoder-decoder, including repetitions of many convolution layers. Specially, this video encoder contains four repetitive convolutional layers with kernel size of $[{k}_{1}^{i},{k}_{2}^{i},{k}_{3}^{i}](i\in [1,4])$, followed by four batch normalizations and ReLU activations, and max poolings with kernel size of $[{s}_{1}^{i},{s}_{2}^{i},{s}_{3}^{i}](i\in [1,4])$, and then generates patches *c*,

2 $$c={{{\bf{g}}}}(\widehat{x}).$$

The convolutional kernel size is ${k}_{1}^{1}={k}_{2}^{1}={k}_{3}^{1}=7$, ${k}_{1}^{2}={k}_{2}^{2}={k}_{3}^{2}=5$, ${k}_{1}^{3}={k}_{2}^{3}={k}_{3}^{3}=3$ and ${k}_{1}^{4}={k}_{2}^{4}={k}_{3}^{4}=3$, and the stride is of 1. The max pooling with kernel size of ${s}_{1}^{1}=3,{s}_{2}^{1}={s}_{3}^{1}=2$, ${s}_{1}^{2}=3,{s}_{2}^{2}={s}_{3}^{2}=2$, ${s}_{1}^{3}=5,{s}_{2}^{3}={s}_{3}^{3}=2$, and ${s}_{1}^{4}=1,{s}_{2}^{4}={s}_{3}^{4}=2$, respectively.

The video decoder, which is a reflection of the encoder, encompasses four convolutional layers, batch normalizations, ReLU activations, and up-samplings. It would then yield an output $v\in {{\mathbb{R}}}^{{{\rm{channels,}}}\,{T,}\,{{\rm{height,}}}\,{{\rm{width}}}}$,

3 $$v={{{\bf{h}}}}(c)$$

where channels, height, and width are 3, 32, and 32, respectively. Here we used an image of 64 × 64 pixels but cropped it into 32 × 32 due to the black background surrounding it (see Supplementary Information). Finally, we employed the hyperbolic tan function to regulate the output $\widehat{v}$ within the range of [0, 1],

4 $$\widehat{v}=(\tanh (v)+1)/2,$$

and

5 $$\tanh (x)=\frac{{e}^{x}-{e}^{-x}}{{e}^{x}+{e}^{-x}},$$

thus elegantly transforming brain data to RGB-based videos.

### Loss function

We formulate the reconstruction task as a regression problem. The loss function employed in our model comprises two components: the Structural Similarity Index Measure (SSIM) that encourages to preservation of structural information, and the Mean Squared Error (MSE) that minimizes the average squared differences between corresponding pixels. This hybrid loss approach is crafted to encompass both the perceptual similarity between images and the pixel-wise intensity differences. Formally, the loss function is as follows:

6 $${{{\rm{Loss}}}}={\sum }_{i=1}^{T}({\eta }_{i}(1-{{{\rm{SSIM}}}}({v}_{g}^{i},{\widehat{v}}^{i})))+{\parallel \!\!{v}_{g}-\widehat{v}\!\!\parallel }_{2}^{2}$$

where ${v}_{g}^{i}$ denotes the *i*-th image in the ground truth video, ${\widehat{v}}^{i}$ indicates the *i*-th image from the reconstructed video, and *η* is the weight assigned to different images. We chose *η* = 1, 2, 5 for fixation, cue, and stimulus images, respectively. For more detailed information about the loss function, please see Supplementary Information.

### Decoding performance

We adopted a training/test ratio of 8:2, randomly split from trial samples. To validate our model performance, we employed 5-fold cross-validation. Specifically, we trained the decoding model on 4 independent folds and tested its performance (measured by MSE and SSIM) on the remaining one fold (Supplementary Tables 1 and 2).

### Encoding model

We inverted the architecture of the decoding model to create the encoding model. Specifically, the encoding model consists of a 3D U-Net (i.e., the components **h** and **g**, with max-pooling first and then up-sampling) followed by a spike decoder **f**. The choice of activation function remains unchanged, the same as the one used in the decoding model. We trained the encoding model by using the training output in the decoding model as input and the training input as output. The encoding results are obtained from the test dataset.

### Evaluating reconstructed videos

In this study, we selected a batch size of 64 and employed the Adamax optimizer with an initial learning rate of 0.01 because of its robustness in handling sparse gradients. The number of training epochs is 40. Additionally, to enhance the training process and achieve better performance, we implemented a StepLR scheduler^50^, which reduced the learning rate by a factor of 0.85 every 3 epochs.

### Cue images

The cue images could be one of four shapes: cross, quatrefoil, circle, or triangle. These cues appeared between 500 ms and 1500 ms after the fixation started at 0 ms. Since the cue in the stimulus video remains static, we directly assessed the Pearson correlation of pixels between the reconstructed and original cue images.

### RGB color of stimulus images

As the stimulus points move in a specific direction over time, color variations occur at consistent pixel positions across frames. Considering the randomized initial positions on each trial, we computed aggregated type-based stimulus images across trials, resulting in stimulus dots arranged within a circle. The RGB values could slightly deviate from the assigned colors due to the black background. Specifically, we selected four symmetric points located at 2/4 and 3/4 positions of the aggregated stimulus images to evaluate the correlation between predicted stimulus dots and the ground truth based on their respective RGB values.

### Movement across stimulus images

To assess the movement of stimulus dots over time, we conducted optical flow calculations on a sequence of images. This entailed iterating through frames, converting each frame image to grayscale, and computing optical flow using the Farneback method. The averaged motion vector was derived from the optical flow matrix over consecutive frames with a step of 2. This algorithm is implemented in OpenCV as calcOpticalFlowFarneback^51^.

### Visualization of high-dimensional data

To illustrate the relationship between spiking activity and visual stimuli, we applied two unsupervised dimensionality reduction techniques: the linear method PCA and the nonlinear method t-SNE^52^. For spiking activity, each sample was represented by a 45-dimensional vector, corresponding to spike counts in 100-ms time bins over 4.5 s stimulus video, and projected to a two-dimensional plane. For model representations, the 512-dimensional latent features, extracted from the intermediate layers of the 3D U-Net (i.e., the blue blocks in Fig. 2A), were reduced to two dimensions for visualization purposes.

### Frequency-based evaluation of spiking activity

To assess how well the model captured temporal properties in spiking activity, we separated spiking activity into low- and high-frequency components and evaluated the prediction score for each. For every brain region, both the ground-truth and model-predicted activity were filtered using a fourth-order Butterworth filter^44^ via the *filtfilt* function from the SciPy^53^. The cutoff frequencies were set at 2 Hz and 10 Hz, corresponding to low (2–10 Hz) and high (>10 Hz) bands, respectively. The *R*^2^ score between filtered ground-truth and predicted activity was then calculated for each frequency band.

### Baseline models

To evaluate the performance of the decoding model (3D U-Net), we implemented two baseline decoders under the same training conditions and hyperparameter settings as the main model. Both models received the same multi-unit spiking activity input and were trained to reconstruct the corresponding 4 cues × 16 color-motion visual stimuli across *T* = 512 trial samples per condition.

Multi-layer perceptron (MLP). The MLP baseline replaced the convolutional decoder with three fully connected layers of 512-1024-3072 units, each followed by batch normalization and ReLU activation. The final layer used a sigmoid activation and was reshaped to match the decoded video frame size. This architecture lacks spatial convolutions and therefore serves as a non-spatial control to test whether frame-level reconstruction can be achieved purely through global feature mapping.

Shallow CNN. The shallow CNN baseline used a lightweight 2D convolutional decoder that operated independently on each temporal snapshot. The input features at each time step were first expanded using a fully connected layer. The decoder then applied two convolutional blocks (each with 3 × 3 kernels, stride 1, and padding 1), each followed by batch normalization and a ReLU activation. The model performed two upsampling operations using bilinear interpolation (scale factor 2) and further refined the output with additional 3 × 3 convolutions before a final sigmoid layer produced the RGB video frames. Unlike the 3D U-Net decoding model, this baseline omitted temporal convolutions and skip connections to isolate the effects of network depth and spatiotemporal context aggregation.

All baseline models were trained under identical optimization settings. The MLP (3,746,484 parameters) was designed to roughly match, but be slightly larger than, our 3D U-Net (3,554,915 parameters), while the shallow CNN contained fewer parameters (602,115). Reconstruction accuracy and correlation metrics were computed identically across all models to ensure a fair comparison, and the results are presented in Supplementary Tables 3 and 4 and Supplementary Fig. 1. Although the MLP and shallow CNN differ architecturally, they share the underlying limitation of lacking spatiotemporal feature pathways, which causes them to converge to similarly coarse approximations and can even lead to mode collapse in individual RGB channels, manifested as negative correlations (Supplementary Fig. 1C).

### Statistics and reproducibility

We evaluated the statistical significance of the comparison results. To validate the function of each individual region learned by the model, we conducted a two-sided *t*-test comparing the predicted pixels reconstructed using masked spiking data to their corresponding predicted pixels without masks. We assumed that the sample distributions of reconstruction errors approximately followed a Gaussian distribution. Additionally, to assess the statistical significance of predicted videos and spiking activity in each individual brain area (PFC, FEF, LIP, MT, IT, and V4), we generated a null distribution from 100 independent predictions using random Gaussian noise inputs drawn from ${{{\mathcal{N}}}}(0,1)$. Specifically, we used random activity as activity input in the decoding process, while random pixels were used as video input in the encoding process. The p-values were computed as the fraction of null sequences with predicted pixels/activities differing from the output (n = 64 stimuli). All *p*-values were indicated as follows: ^*^p < 0.05, ^**^p < 0.01, ^***^p < 0.001, ^****^p < 0.0001. All results were analyzed using the monkey MUA dataset^24^, and no biological replicates were used in the statistical analyses. Model-based analyses were conducted with fixed random seeds to ensure computational reproducibility.

### Reporting summary

Further information on research design is available in the Nature Portfolio Reporting Summary linked to this article.

## Supplementary information

Supplementary Information

Description of Additional Supplementary Files

Supplementary Data 1

Supplementary Movie 1

Supplementary Movie 2

Supplementary Movie 3

Reporting summary

## Data Availability

Source data are provided with this paper in the Supplementary Data 1 file.

## Code availability

The codes used in this work are available at Zenodo^54^.

## References

[1] Richards, B. A. et al. A deep learning framework for neuroscience. *Nat. Neurosci.* **22**, 1761–1770 (2019). 31659335 10.1038/s41593-019-0520-2 PMC7115933

[2] Saxe, A., Nelli, S. & Summerfield, C. If deep learning is the answer, what is the question?. *Nat. Rev. Neurosci.* **22**, 55–67 (2021). 33199854 10.1038/s41583-020-00395-8

[3] Kriegeskorte, N. Deep neural networks: a new framework for modeling biological vision and brain information processing. *Annu. Rev. Vis. Sci.* **1**, 417–446 (2015). 28532370 10.1146/annurev-vision-082114-035447

[4] Serre, T. Deep learning: the good, the bad, and the ugly. *Annu. Rev. Vis. Sci.* **5**, 399–426 (2019). 31394043 10.1146/annurev-vision-091718-014951

[5] Yu, Q. et al. Visual cortex encodes timing information in humans and mice. *Neuron* **110**, 4194–4211 (2022). 36195097 10.1016/j.neuron.2022.09.008

[6] Loriette, C., Amengual, J. L. & Ben Hamed, S. Beyond the brain-computer interface: Decoding brain activity as a tool to understand neuronal mechanisms subtending cognition and behavior. *Front. Neurosci.* **16**, 811736 (2022). 36161174 10.3389/fnins.2022.811736 PMC9492914

[7] Farzmahdi, A., Zarco, W., Freiwald, W. A., Kriegeskorte, N. & Golan, T. Emergence of brain-like mirror-symmetric viewpoint tuning in convolutional neural networks. *eLife* **13**, e90256 (2024). 38661128 10.7554/eLife.90256 PMC11142642

[8] Wu, S. et al. Neural heterogeneity enhances reliable neural information processing: Local sensitivity and globally input-slaved transient dynamics. *Sci. Adv.* **11**, eadr3903 (2025). 40173217 10.1126/sciadv.adr3903 PMC11963962

[9] Deco, G. et al. The arrow of time of brain signals in cognition: Potential intriguing role of parts of the default mode network. *Netw. Neurosci.* **7**, 966–998 (2023). 37781151 10.1162/netn_a_00300 PMC10473271

[10] Tatsukawa, T. & Teramae, J. -n The cortical critical power law balances energy and information in an optimal fashion. *Proc. Natl. Acad. Sci. U.S.A.* **122**, e2418218122 (2025). 40408401 10.1073/pnas.2418218122 PMC12130854

[11] Chen, Y., Wang, S., Hilgetag, C. C. & Zhou, C. Trade-off between multiple constraints enables simultaneous formation of modules and hubs in neural systems. *PLoS Comput. Biol.* **9**, e1002937 (2013). 23505352 10.1371/journal.pcbi.1002937 PMC3591279

[12] Yamins, D. L. & DiCarlo, J. J. Using goal-driven deep learning models to understand sensory cortex. *Nat. Neurosci.* **19**, 356–365 (2016). 26906502 10.1038/nn.4244

[13] Zhang, Y. et al. Decoding of human identity by computer vision and neuronal vision. *Sci. Rep.* **13**, 651 (2023). 36635322 10.1038/s41598-022-26946-w PMC9837190

[14] Lindsay, G. W. Convolutional neural networks as a model of the visual system: Past, present, and future. *J. Cogn. Neurosci.* **33**, 2017–2031 (2021). 32027584 10.1162/jocn_a_01544

[15] Tang, J., LeBel, A., Jain, S. & Huth, A. G. Semantic reconstruction of continuous language from non-invasive brain recordings. *Nat. Neurosci*. 1–9 (2023). 10.1038/s41593-023-01304-9 PMC11304553 37127759

[16] Stoyanovich, J., Van Bavel, J. J. & West, T. V. The imperative of interpretable machines. *Nat. Mach. Intell.* **2**, 197–199 (2020).

[17] Xu, Y. & Vaziri-Pashkam, M. Limits to visual representational correspondence between convolutional neural networks and the human brain. *Nat. Commun.* **12**, 2065 (2021). 33824315 10.1038/s41467-021-22244-7 PMC8024324

[18] Kay, K. N. Principles for models of neural information processing. *NeuroImage* **180**, 101–109 (2018). 28793238 10.1016/j.neuroimage.2017.08.016

[19] Dong, H.-W. et al. Neural networks of the mouse primary visceromotor cortex. *Research Square* rs–3 (2025). 10.1038/s41586-025-09360-w 40866707

[20] Mack, M. L., Preston, A. R. & Love, B. C. Ventromedial prefrontal cortex compression during concept learning. *Nat. Commun.* **11**, 46 (2020). 31911628 10.1038/s41467-019-13930-8 PMC6946809

[21] Mack, M. L., Love, B. C. & Preston, A. R. Dynamic updating of hippocampal object representations reflects new conceptual knowledge. *Proc. Natl. Acad. Sci. U.S.A.* **113**, 13203–13208 (2016). 27803320 10.1073/pnas.1614048113 PMC5135299

[22] Srivastava, S., Wang, W. Y. & Eckstein, M. P. Emergent human-like covert attention in feedforward convolutional neural networks. *Curr. Biol.* **34**, 579–593 (2024). 38244541 10.1016/j.cub.2023.12.058

[23] Zhang, X.-Y. et al. Adaptive stretching of representations across brain regions and deep learning model layers. *Nat. Commun.* **16**, 10302 (2025). 41271767 10.1038/s41467-025-65231-y PMC12638828

[24] Siegel, M., Buschman, T. J. & Miller, E. K. Cortical information flow during flexible sensorimotor decisions. *Science* **348**, 1352–1355 (2015). 26089513 10.1126/science.aab0551 PMC4721574

[25] Yoshida, T. & Ohki, K. Natural images are reliably represented by sparse and variable populations of neurons in visual cortex. *Nat. Commun.* **11**, 872 (2020). 32054847 10.1038/s41467-020-14645-x PMC7018721

[26] Zurawel, G., Shamir, I. & Slovin, H. Reconstruction of shape contours from v1 activity at high resolution. *NeuroImage* **125**, 1005–1012 (2016). 26518630 10.1016/j.neuroimage.2015.10.072

[27] Kobatake, E. & Tanaka, K. Neuronal selectivities to complex object features in the ventral visual pathway of the macaque cerebral cortex. *J. Neurophysiol.* **71**, 856–867 (1994). 8201425 10.1152/jn.1994.71.3.856

[28] Born, R. T. & Bradley, D. C. Structure and function of visual area MT. *Annu. Rev. Neurosci.* **28**, 157–189 (2005). 16022593 10.1146/annurev.neuro.26.041002.131052

[29] Simoncelli, E. P. & Heeger, D. J. A model of neuronal responses in visual area MT. *Vis. Res.* **38**, 743–761 (1998). 9604103 10.1016/s0042-6989(97)00183-1

[30] Miller, E. K. & Cohen, J. D. An integrative theory of prefrontal cortex function. *Annu. Rev. Neurosci.* **24**, 167–202 (2001). 11283309 10.1146/annurev.neuro.24.1.167

[31] Haynes, J.-D. & Rees, G. Decoding mental states from brain activity in humans. *Nat. Rev. Neurosci.* **7**, 523–534 (2006). 16791142 10.1038/nrn1931

[32] Kamitani, Y. & Tong, F. Decoding the visual and subjective contents of the human brain. *Nat. Neurosci.* **8**, 679–685 (2005). 15852014 10.1038/nn1444 PMC1808230

[33] Kamitani, Y. & Tong, F. Decoding seen and attended motion directions from activity in the human visual cortex. *Curr. Biol.* **16**, 1096–1102 (2006). 16753563 10.1016/j.cub.2006.04.003 PMC1635016

[34] Khaligh-Razavi, S.-M. & Kriegeskorte, N. Deep supervised, but not unsupervised, models may explain it cortical representation. *PLoS Comput. Biol.* **10**, e1003915 (2014). 25375136 10.1371/journal.pcbi.1003915 PMC4222664

[35] Yamins, D. L. et al. Performance-optimized hierarchical models predict neural responses in higher visual cortex. *Proc. Natl. Acad. Sci. U.S.A.* **111**, 8619–8624 (2014). 24812127 10.1073/pnas.1403112111 PMC4060707

[36] Horikawa, T. & Kamitani, Y. Generic decoding of seen and imagined objects using hierarchical visual features. *Nat. Commun.* **8**, 15037 (2017). 28530228 10.1038/ncomms15037 PMC5458127

[37] Scotti, P. S. et al. Mindeye2: Shared-subject models enable fmri-to-image with 1 hour of data. *ICLR Workshop on Representational Alignment* (2024).

[38] Pasupathy, A. & Connor, C. E. Population coding of shape in area v4. *Nat. Neurosci.* **5**, 1332–1338 (2002). 12426571 10.1038/nn972

[39] Newsome, W. T., Britten, K. H. & Movshon, J. A. Neuronal correlates of a perceptual decision. *Nature* **341**, 52–54 (1989). 2770878 10.1038/341052a0

[40] Shadlen, M. N. & Newsome, W. T. Neural basis of a perceptual decision in the parietal cortex (area lip) of the rhesus monkey. *J. Neurophysiol.* **86**, 1916–1936 (2001). 11600651 10.1152/jn.2001.86.4.1916

[41] Sarma, A., Masse, N. Y., Wang, X.-J. & Freedman, D. J. Task-specific versus generalized mnemonic representations in parietal and prefrontal cortices. *Nat. Neurosci.* **19**, 143–149 (2016). 26595652 10.1038/nn.4168 PMC4880358

[42] Atanas, A. A. et al. Brain-wide representations of behavior spanning multiple timescales and states in c. elegans. *Cell* **186**, 4134–4151 (2023). 37607537 10.1016/j.cell.2023.07.035 PMC10836760

[43] Beguš, G., Zhou, A. & Zhao, T. C. Encoding of speech in convolutional layers and the brain stem based on language experience. *Sci. Rep.* **13**, 6480 (2023). 37081119 10.1038/s41598-023-33384-9 PMC10119295

[44] Butterworth, S. et al. On the theory of filter amplifiers. *Wireless Engr.* **7**, 536–541 (1930).

[45] Yu, Z. & Huang, H. Nonequilbrium physics of generative diffusion models. *Phys. Rev. E* **111**, 014111 (2025). 39972748 10.1103/PhysRevE.111.014111

[46] Zhang, X.-Y., Moore, J. M., Ru, X. & Yan, G. Geometric scaling law in real neuronal networks. *Phys. Rev. Lett.* **133**, 138401 (2024). 39392951 10.1103/PhysRevLett.133.138401

[47] Yang, Z., Liang, J. & Zhou, C. Critical avalanches in excitation-inhibition balanced networks reconcile response reliability with sensitivity for optimal neural representation. *Phys. Rev. Lett.* **134**, 028401 (2025). 39913846 10.1103/PhysRevLett.134.028401

[48] Cowley, B. R. et al. Mapping model units to visual neurons reveals population code for social behaviour. *Nature* **629**, 1100–1108 (2024). 38778103 10.1038/s41586-024-07451-8 PMC11136655

[49] Matsugu, M., Mori, K., Mitari, Y. & Kaneda, Y. Subject independent facial expression recognition with robust face detection using a convolutional neural network. *Neural Netw.* **16**, 555–559 (2003). 12850007 10.1016/S0893-6080(03)00115-1

[50] Kim, C., Kim, S., Kim, J., Lee, D. & Kim, S. Automated learning rate scheduler for large-batch training. In *ICML Workshop on Automated Machine Learning* (ICML, 2021).

[51] Farnebäck, G. Two-frame motion estimation based on polynomial expansion. In *Image Analysis* 363–370 (Springer, 2003).

[52] Van der Maaten, L. & Hinton, G. Visualizing data using t-sne. *J. Mach. Learn. Res*. **9**, 2579–2605 (2008).

[53] Virtanen, P. et al. SciPy 1.0: fundamental algorithms for scientific computing in python. *Nat. Methods* **17**, 261–272 (2020). 32015543 10.1038/s41592-019-0686-2 PMC7056644

[54] Zhang, X.-Y. Neuraldecoding: Code for *Data-driven ANN-based visual decoding enables unsupervised functional alignment*. Zenodo 10.5281/zenodo.17845924 (2025). 10.1038/s42003-025-09486-7 PMC12894685 41507464

