# Supplementary material for: Data-driven ANN-based visual decoding enables unsupervised functional alignment
Source: Commun Biol. 2026 Jan 8;9:210. doi: 10.1038/s42003-025-09486-7 (PMC12894685; doi:10.1038/s42003-025-09486-7)
Supplement: Supplementary file 1 — Supplementary Information [file 42003_2025_9486_MOESM1_ESM.pdf]

Supplementary Information for

Data-driven ANN-based visual decoding enables unsupervised functional alignment

Xin-Ya Zhang, Hang Lin, Zeyu Deng, Markus Siegel, Earl K. Miller, Gang Yan

Contents

Supplementary Note 1: Stimulus image **2**

Supplementary Note 2: Decoding model **2**

Supplementary Note 3: Estimating optical flow **3**

Supplementary Note 4: Visualization of high-dimensional data **4**

Supplementary Tables **5**

Supplementary Figures **11**

Supplementary References **13**

Supplementary Note 1: Stimulus image

Stimulus images were 64×64 RGB pixels. In the fixation image, the distance between the fixation point and either of the grey dots is equal to 1/4 of the horizontal length of the image. This ratio is kept constant for the rest of the image types. Monochrome cue images were imported, the cue color changed to grey, the background to black, and three dots (also present in the fixation point image) added. The stimuli are dynamic random dot patterns with 100% motion coherence, centered on the fixation spot. The stimuli have a diameter of 3.2, a dot diameter of 0.08, 400 dots, and two dot speeds (1.67 /s or 10 /s) for half of the recording trials. We considered four possible colors and four possible directions for the stimulus dots. The direction labels are $-90°, -30°, 30°, 90°$. All colors are defined in the CIE Lab* space with identical luminance and saturation. In total, there were 16 possible color-motion combinations presented in Fig. 1. Due to the redundant black background in the video surroundings, we cropped the 64×64 pixels to 32×32 pixels by removing the surrounding black background.

Supplementary Note 2: Decoding model

To train our decoding model, we replicated fixation and cue images from the original experiment and generated stimulus images with the above-mentioned stimulus patterns. As the fixation point and cue images were static, we repeated them to simulate the movies presented to the monkeys. Considering an f-ms window for processing MUA data (equivalent to a frequency of $1/f$ frames per second), each trial comprises $4.5 \times 1/f$ images, including $0.5 \times 1/f$ fixation point images, $1.0 \times 1/f$ cue images, and $3.0 \times 1/f$ stimulus images. In this study, the time bin/frequency parameter f was selected to ensure an integer result for the specified number of images mentioned above.

Loss function

The loss function employed in our model comprises two components: the Structural Similarity Index Measure (SSIM) and the Mean Squared Error (MSE). For any images x and y, the objective function is to minimize

$$\text{Loss} = \{1 - \text{SSIM}(x, y), \text{MSE}(x, y)\}. \tag{S1}$$

The SSIM component encourages the model to preserve structural information. For images x and y, SSIM is calculated using the following formula:

$$\mathrm{SSIM}(x,y) = \frac{(2\mu_x\mu_y + c_1)(2\sigma_{xy} + c_2)}{(\mu_x^2 + \mu_y^2 + c_1)(\sigma_x^2 + \sigma_y^2 + c_2)} \tag{S2}$$

where μ_x and μ_y represent mean intensities of images x and y, σ_x^2 and σ_y^2 are the variances of x and y, σ_{xy} denotes the covariance between x and y, and c_1, c_2 are the constants to avoid instability near zero. The SSIM ranges from -1 to 1, where 1 indicates perfect similarity between images. Therefore, we utilized $\eta \cdot (1 - \mathrm{SSIM}(x,y))$ in our study, where η denotes the importance weight assigned to different images. Specifically, we set η to 1, 2, and 5 for fixation, cue, and stimulus images, respectively.

The MSE term ensures that the model minimizes the mean squared differences between corresponding pixels,

$$\mathrm{MSE}(x,y) = \|x - y\|_2^2 \tag{S3}$$

thereby increasing the overall color accuracy of the reconstructed images.

Supplementary Note 3: Estimating optical flow

To compute the optical flow, we employed the Farneback method. Initially, we iterated through each frame of the video, converting it to grayscale using the OpenCV `cvtColor` function. The optical flow was then computed using the Farneback method, implemented through the `calcOpticalFlowFarneback` function in OpenCV [1],

$$\begin{aligned} \mathit{flow} = \ & \mathrm{calcOpticalFlowFarneback}(\mathit{prev}, \mathit{next}, \\ & \mathit{pyr\_scale}, \mathit{levels}, \mathit{winsize}, \mathit{iterations}, \mathit{poly\_n}, \mathit{poly\_sigma}, \mathit{flags}) \end{aligned} \tag{S4}$$

where *prev* and *next* represent the input frames (i.e., the grayscale images), and *flow* denotes the resulting optical flow vector. The algorithm parameters, including *pyr_scale*, *levels*, *winsize*, *iterations*, *poly_n*, *poly_sigma*, *flags*, are set to 0.5, 3, 15, 3, 5, 1.2, and 0, respectively.

Finally, we obtained the averaged motion vector from the optical flow matrix over consecutive frames with a specified step (e.g., a step of 2). Through these steps, we successfully

applied the Farneback method to calculate the optical flow of objects within a video.

Supplementary Note 4: Visualization of high-dimensional data

We utilized two unsupervised dimensionality reduction methods: Principal Component Analysis (PCA) and t-Distributed Stochastic Neighbor Embedding (t-SNE).

Principal Component Analysis. PCA was conducted using the `PCA` module from the `scikit-learn` library, with the target dimensionality set to two. PCA results served as a linear baseline for comparison with the nonlinear embedding obtained from t-SNE.

T-distributed stochastic neighbor embedding. The visualization t-SNE is implemented using the `TSNE` module from the `scikit-learn` library, with the output dimensionality set to two and a maximum of 10^3 iterations.

Supplementary Tables

5-fold cross-validation

Supplementary Table 1: k-fold cross-validation in vision decoding. Here we used 5-fold cross-validation, where 4 folds are used to train the decoding model, and 1 fold is used for testing. The structural similarity between reconstructed images and ground truth (SSIM, Eq. S2) is presented.

SSIM<br>k-fold (k=5)	1	2	3	4	5
Train dataset	0.801	0.799	0.798	0.797	0.798
Test dataset	0.773	0.779	0.784	0.779	0.784

Supplementary Table 2: k-fold cross-validation in vision decoding. Here we used 5-fold cross-validation, where 4 folds are used to train the decoding model, and 1 fold is used for testing. The mean squared error between reconstructed images and ground truth (MSE, Eq. S3) is presented.

MSE<br>k-fold (k=5)	1	2	3	4	5
Train dataset	0.0034	0.0035	0.0036	0.0036	0.0035
Test dataset	0.0053	0.0047	0.0039	0.0045	0.0041

Baselines comparison

Supplementary Table 3: Comparison of reconstruction performance across different models. Structural similarity index measure (SSIM, larger is better) is reported for both train and test datasets, showing that the proposed decoding model outperforms the multilayer perceptron (MLP) and shallower CNN baselines.

SSIM (larger is better)	3D U-Net (proposed decoding model)	MLP (baseline)	Shallower CNN (baseline)
Train dataset	0.799	0.733	0.691
Test dataset	0.780	0.706	0.685

Supplementary Table 4: Comparison of reconstruction errors across different decoding models. Mean squared error (MSE, smaller is better) is reported, demonstrating that the proposed decoding model achieves lower reconstruction error than the multilayer perceptron (MLP) and shallower CNN baselines.

MSE (smaller is better)	3D U-Net (proposed decoding model)	MLP (baseline)	Shallower CNN (baseline)
Train dataset	0.0035	0.0104	0.0163
Test dataset	0.0045	0.0137	0.0168

Statistics for masked regions in Fig. 4

Supplementary Table 5: The difference (mean±s.d.) of pixels for cue images reconstructed from data with masked brain regions. A larger value indicates that masking the specific region has a greater impact on cue reconstruction. A two-sided t-test was performed to compare the predicted pixels reconstructed with the masked region to their corresponding unmasked predicted pixels.

Masked region (cue)	mean	s.d.	t-test
PFC	0.0584	0.0583	t(126)=-0.0171, p=0.9864
FEF	0.0832	0.1032	t(126)=0.4191, p=0.6758
LIP	7.1086	4.0081	t(126)=-12.85, p<0.0001
MT	4.7556	2.8706	t(126)=-11.99, p<0.0001
IT	1.8100	1.5199	t(126)=-7.424, p<0.0001
V4	16.416	7.8273	t(126)=-16.34, p<0.0001

Supplementary Table 6: The RGB color difference (mean±s.d.) of pixels for stimulus images reconstructed from data with masked brain regions. A larger value indicates that masking the specific region has a greater impact on the reconstruction of stimulus color. A two-sided t-test was performed to compare the predicted pixels reconstructed with the masked region to their corresponding unmasked predicted pixels.

Masked region (color)	mean	s.d.	t-test
PFC	0.5303	0.4009	t(126)=-0.0792, p=0.9370
FEF	0.4971	0.3969	t(126)=-0.0401, p=0.9681
LIP	8.5693	8.5238	t(126)=-3.0717, p=0.0026
MT	2.9801	2.3269	t(126)=0.8859, p=0.3774
IT	1.0712	0.9326	t(126)=-0.1212, p=0.9037
V4	3.5544	2.6479	t(126)=0.3533, p=0.7245

Supplementary Table 7: The movement angle difference (mean±s.d.) across frames (i.e., stimulus images) reconstructed from data with masked brain regions. A larger value indicates that masking the specific region has a greater impact on the reconstruction of stimulus motion. A two-sided t-test was performed to compare the predicted pixels reconstructed with the masked region to their corresponding unmasked predicted pixels.

Masked region (motion)	mean	s.d.	t-test
PFC	0.9892	0.8283	t(126)=-0.4301, p=0.6678
FEF	0.7911	0.6103	t(126)=0.0993, p=0.9210
LIP	9.183	9.063	t(126)=-5.532, p<0.0001
MT	30.75	40.48	t(126)=-5.711, p<0.0001
IT	2.677	2.792	t(126)=-1.590, p=0.1144
V4	15.88	12.61	t(126)=-8.456, p<0.0001

Multiple-comparison correction in Fig. 4

Supplementary Table 8: Statistical comparison of masking region V4 versus other brain regions for cue shape decoding. We performed t-tests with multiple-comparison correction using false discovery rate (FDR). Significant differences (p-value < 0.5) are indicated in the last column.

Cue shape	t-statistic	p-uncorrected	p-FDR	Significant?
V4 vs. PFC	t(126)=16.586	1.14e-24	3.07e-24	Yes
V4 vs. FEF	t(126)=16.560	1.22e-24	3.07e-24	Yes
V4 vs. LIP	t(126)=8.396	4.70e-13	4.71e-13	Yes
V4 vs. MT	t(126)=11.098	7.73e-18	9.66e-18	Yes
V4 vs. IT	t(126)=14.537	1.65e-22	2.74e-22	Yes

Supplementary Table 9: Pairwise statistical comparison of masking LIP versus other brain regions for color decoding. T-statistics, uncorrected p-values, FDR-corrected p-values, and significance (p-value< 0.5) are shown.

Stimulus color	t-statistic	p-uncorrected	p-FDR	Significant?
LIP vs. PFC	t(126)=7.497	2.59e-10	6.48e-10	Yes
LIP vs. FEF	t(126)=7.632	1.52e-10	6.48e-10	Yes
LIP vs. MT	t(126)=3.159	2.17e-3	2.18e-3	Yes
LIP vs. IT	t(126)=6.410	1.62e-8	2.71e-8	Yes
LIP vs. V4	t(126)=3.693	3.92e-4	4.90e-4	Yes

Supplementary Table 10: Comparison of masking MT against other brain regions for motion decoding performance. The table reports t-statistics, p-values before and after FDR correction, and whether the difference is statistically significant (p-value< 0.5).

Stimulus motion	t-statistic	p-uncorrected	p-FDR	Significant?
MT vs. PFC	t(126)=6.057	8.47e-8	2.11e-7	Yes
MT vs. FEF	t(126)=6.099	7.18e-8	2.11e-7	Yes
MT vs. LIP	t(126)=4.301	5.41e-5	6.76e-5	Yes
MT vs. IT	t(126)=5.745	2.78e-7	4.64e-7	Yes
MT vs. V4	t(126)=3.290	1.54e-3	1.54e-3	Yes

Statistics for individual regions in Fig. 5

Supplementary Table 11: Pearson correlation (mean±s.d.) of pixels between the ground truth and reconstructed cues from individual brain region data. A lower correlation value implies that information from this particular region is less conducive to achieving good performance. To assess the statistical significance, 100 independent tests were conducted using random Gaussian input, and p-values were computed as the fraction of null sequences with predicted pixels differing from random distributions.

Region (cue)	mean	s.d.	t-test
PFC	0.6544	0.2273	$t(162)=6.0369$, $p<0.0001$
FEF	0.6436	0.0927	$t(162)=9.2237$, $p<0.0001$
LIP	0.9986	0.0004	$t(162)=585.9$, $p<0.0001$
MT	0.9985	0.0005	$t(162)=706.1$, $p<0.0001$
IT	0.9977	0.0010	$t(162)=842.5$, $p<0.0001$
V4	0.9986	0.0004	$t(162)=755.8$, $p<0.0001$

Supplementary Table 12: Pearson correlation (mean±s.d.) of RGB values between the ground truth and reconstructed stimulus images from individual brain region data. A lower correlation value implies that information from this particular region is less conducive to achieving good performance. To assess the statistical significance, 100 independent tests were conducted using random Gaussian input, and p-values were computed as the fraction of null sequences with predicted RGB values differing from random distributions.

Region (color)	mean	s.d.	t-test
PFC	0.8008	0.0261	$t(162)=51.5067$, $p<0.0001$
FEF	0.5826	0.1352	$t(162)=-21.7066$, $p<0.0001$
LIP	0.9967	0.0023	$t(162)=198.0$, $p<0.0001$
MT	0.9997	0.0004	$t(162)=170.9$, $p<0.0001$
IT	0.9930	0.0029	$t(162)=226.1$, $p<0.0001$
V4	0.9982	0.0011	$t(162)=119.8$, $p<0.0001$

Supplementary Table 13: The movement angle difference (mean±s.d.) between the ground truth and reconstructed stimulus videos from individual brain region data. A higher difference value implies that information from this particular region is less conducive to achieving good performance. To assess the statistical significance, 100 independent tests were conducted using random Gaussian input, and p-values were computed as the fraction of null sequences with predicted motion differing from random distributions.

Region (motion)	mean	s.d.	*t*-test
PFC	33.01	25.32	$t(162)=-10.61$, $p<0.0001$
FEF	40.25	34.88	$t(162)=-5.552$, $p<0.0001$
LIP	2.739	2.334	$t(162)=-244.6$, $p<0.0001$
MT	1.882	1.518	$t(162)=-134.4$, $p<0.0001$
IT	4.069	2.912	$t(162)=-207.8$, $p<0.0001$
V4	2.041	1.603	$t(162)=-330.1$, $p<0.0001$

Statistics for brain encoding in Fig. 6

Supplementary Table 14: Pearson correlation (mean±s.d.) between the recorded activity and the activity predicted from the inverse decoding model. A higher correlation value indicates better performance in predicting activity within that region. To assess the statistical significance, 100 independent tests were conducted using random Gaussian input, and p-values were computed as the fraction of null sequences with predicted activity differing from random distributions.

Encoding	mean	s.d.	*t*-test
PFC	0.5146	0.0880	$t(162)=7.624$, $p<0.0001$
FEF	0.5476	0.0929	$t(162)=2.078$, $p=0.0391$
LIP	0.7671	0.0932	$t(162)=15.43$, $p<0.0001$
MT	0.9016	0.0204	$t(162)=14.09$, $p<0.0001$
IT	0.8772	0.0280	$t(162)=47.12$, $p<0.0001$
V4	0.9352	0.0110	$t(162)=80.28$, $p<0.0001$

Supplementary Figures

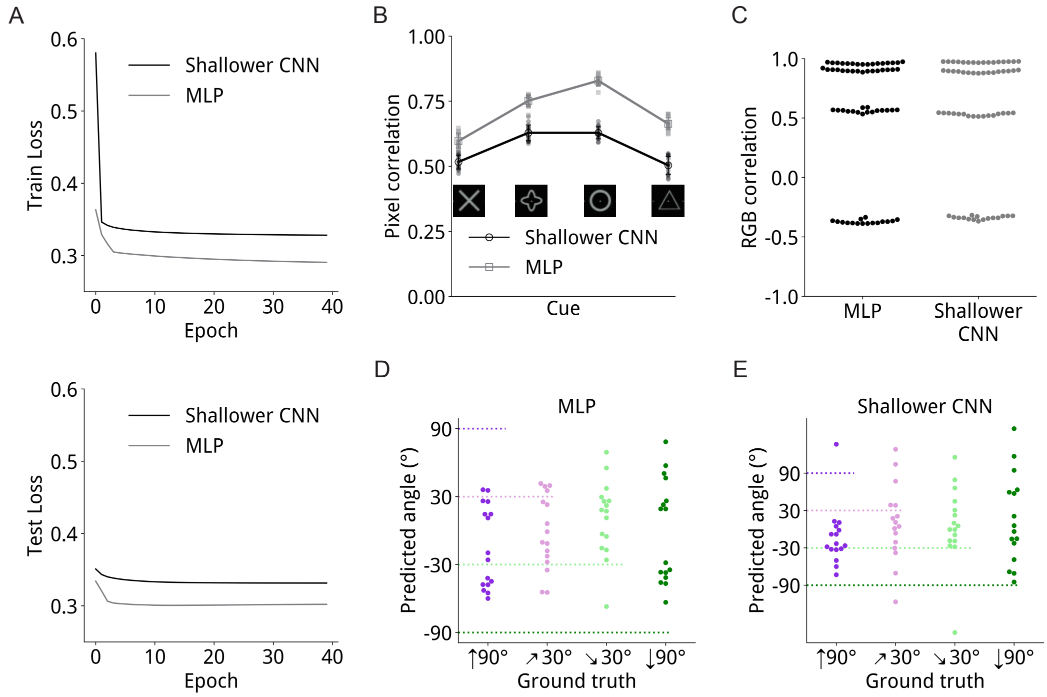

Supplementary Figure 1: **Baseline model performance.** (**A**) Train and test loss curves for the MLP and shallow CNN baseline decoders over training epochs. Both curves converge to stable values, indicating neither overfitting nor underfitting. (**B**) Pixel-level correlation between reconstructed and ground-truth frames ($n = 16$ color-motion samples) for the MLP and shallow CNN baseline decoders across different cue conditions. (**C**) RGB-channel correlations of reconstructed frames ($n = 64$ independent stimulus samples), showing that both baseline models lack fine-grained fidelity compared with the 3D U-Net decoder (Fig. 2). (**D-E**) Predicted versus ground-truth motion directions for the MLP (D) and shallow CNN (E) decoders. Each dot represents one stimulus condition ($n = 64$ independent stimulus samples in total), and dashed lines indicate the correct direction angles. Both baseline models tend to misestimate motion direction. The baseline models exhibit discrete and negative RGB correlations because of limited spatiotemporal capacity and resulting channel collapse, whereas the 3D U-Net's spatiotemporal convolutions integrate space and time to avoid such effects.

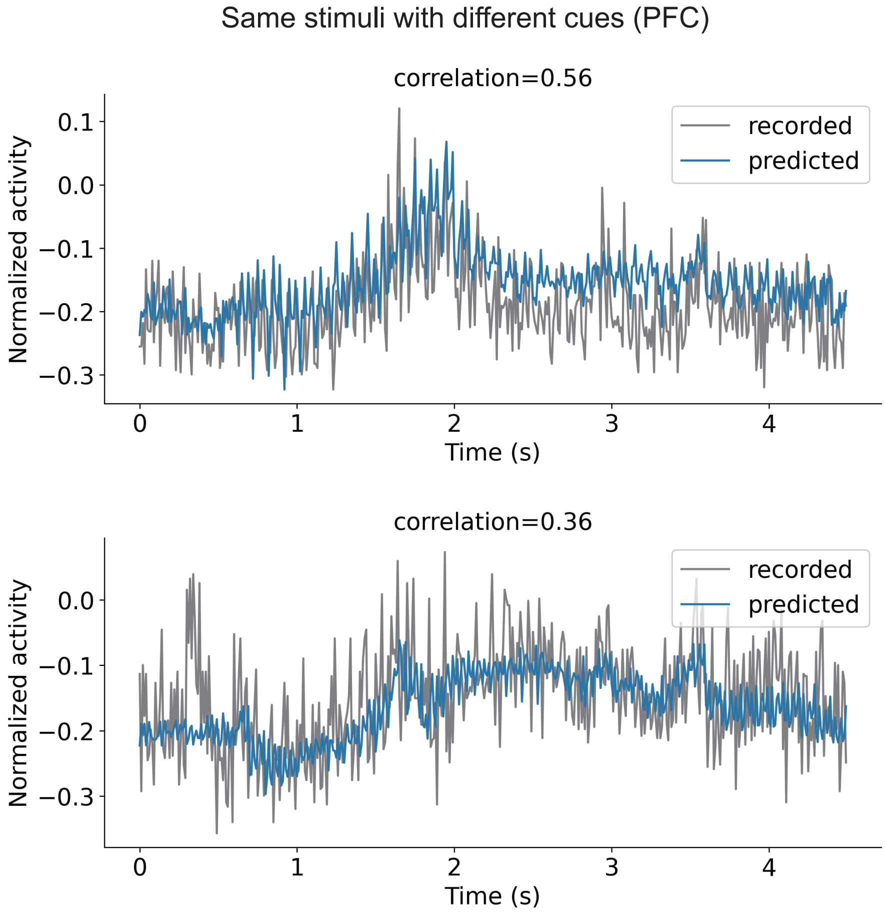

Supplementary Figure 2: **Examples of recorded and predicted activities in PFC showing low correlations.** Top and bottom panels show recorded (gray) and predicted (blue) spiking activities in the prefrontal cortex (PFC) for a representative stimulus sample when the monkey was presented with the same stimuli but different cues. Compared with the IT region shown in Fig. 6, PFC recordings exhibit stronger high-frequency fluctuations, suggesting more variable and less stimulus-locked neural responses.

Supplementary References

[1] Farnebäck, G. Two-frame motion estimation based on polynomial expansion. In *Image Analysis*, 363–370 (Springer, 2003).
