# Supplementary material for: Data-driven ANN-based visual decoding enables unsupervised functional alignment
Source: Commun Biol. 2026 Jan 8;9:210. doi: 10.1038/s42003-025-09486-7 (PMC12894685; doi:10.1038/s42003-025-09486-7)
Supplement: Supplementary file 2 — Description of Additional Supplementary Files [file 42003_2025_9486_MOESM2_ESM.pdf]

Description of Additional Supplementary File

File name: Supplementary Data 1

Description: Source data are provided with this paper in the Supplementary Data file

File name: Supplementary Movie 1

Description: The supplementary movie for 50 ms frequency.

File name: Supplementary Movie 2

Description: The supplementary movie for 100 ms frequency.

File name: Supplementary Movie 3

Description: The supplementary movie for 100 ms frequency with region masks.
